# Cardiac Development in Zebrafish and Human Embryonic Stem Cells Is Inhibited by Exposure to Tobacco Cigarettes and E-Cigarettes

**DOI:** 10.1371/journal.pone.0126259

**Published:** 2015-05-15

**Authors:** Nathan J. Palpant, Peter Hofsteen, Lil Pabon, Hans Reinecke, Charles E. Murry

**Affiliations:** 1 Department of Pathology, University of Washington School of Medicine, Seattle, Washington, United States of America; 2 Department of Bioengineering, University of Washington School of Medicine, Seattle, Washington, United States of America; 3 Department of Medicine/Cardiology, University of Washington School of Medicine, Seattle, Washington, United States of America; 4 Center for Cardiovascular Biology, University of Washington School of Medicine, Seattle, Washington, United States of America; 5 Institute for Stem Cell and Regenerative Medicine, University of Washington School of Medicine, Seattle, Washington, United States of America; Northwestern University, UNITED STATES

## Abstract

**Background:**

Maternal smoking is a risk factor for low birth weight and other adverse developmental outcomes.

**Objective:**

We sought to determine the impact of standard tobacco cigarettes and e-cigarettes on heart development *in vitro* and *in vivo*.

**Methods:**

Zebrafish (*Danio rerio*) were used to assess developmental effects *in vivo* and cardiac differentiation of human embryonic stem cells (hESCs) was used as a model for *in vitro* cardiac development.

**Results:**

In zebrafish, exposure to both types of cigarettes results in broad, dose-dependent developmental defects coupled with severe heart malformation, pericardial edema and reduced heart function. Tobacco cigarettes are more toxic than e-cigarettes at comparable nicotine concentrations. During cardiac differentiation of hESCs, tobacco smoke exposure results in a delayed transition through mesoderm. Both types of cigarettes decrease expression of cardiac transcription factors in cardiac progenitor cells, suggesting a persistent delay in differentiation. In definitive human cardiomyocytes, both e-cigarette- and tobacco cigarette-treated samples showed reduced expression of sarcomeric genes such as MLC2v and MYL6. Furthermore, tobacco cigarette-treated samples had delayed onset of beating and showed low levels and aberrant localization of N-cadherin, reduced myofilament content with significantly reduced sarcomere length, and increased expression of the immature cardiac marker smooth muscle alpha-actin.

**Conclusion:**

These data indicate a negative effect of both tobacco cigarettes and e-cigarettes on heart development *in vitro* and *in vivo*. Tobacco cigarettes are more toxic than E-cigarettes and exhibit a broader spectrum of cardiac developmental defects.

## Introduction

Maternal smoking is one of the most significant causes of preventable perinatal morbidity and mortality worldwide, accounting for nearly $200 billion in health care costs each year within the United States alone[1]. Smoking increases the risk of a wide range of pregnancy complications including miscarriage. Studies have shown that maternal smoking is strongly associated with placental developmental deficits, ectopic implantation, stillbirth, reduced birth weight, structural malformations as well as systemic disorders including postnatal neurobehavioral and cardiovascular problems and increased risk of Sudden Infant Death Syndrome (SIDS)[2–4].

Tobacco cigarette smoke has over 7,000 chemicals, including at least 69 known carcinogens such as tar, heavy metals, polycyclic aromatic hydrocarbons, phenol, benzene, carbon monoxide, nitrosamines, and hydrogen cyanide distributed throughout the gas and particulate phases [4]. The complexity of these chemical components and their interactions with each other in the context of human physiology are not well understood. Among the chemicals in tobacco smoke, nicotine is without question the most widely studied and understood bioactive agent [4]. During pregnancy, nicotine and other metabolites diffuse across the chorioamnionic membrane into fetal serum, where the low pH of amniotic fluid favors the accumulation of basic metabolites like nicotine in the fetus [5]. In accordance with this, studies have shown that fetal nicotine levels are higher than maternal serum and tend to accumulate in amniotic fluid, fetal serum and meconium [6, 7].

Electronic cigarettes (e-cigarettes) are a recent technology that allows for nicotine delivery without the complex components of tobacco cigarette. E-cigarettes contain a cartridge with a humectant mixed with a variety of artificial flavorings and nicotine that is aerosolized by heat generated by an atomizer. Based on the chemical composition of e-cigarettes (containing fewer chemicals including the wide range of known carcinogens found in traditional cigarettes), they are gaining significant popularity in recent years [8–10], and currently there is little oversight or regulation over their use and sale[11]. More research is necessary to determine the consequences of e-cigarettes on pregnancy or development.

A variety of cell culture and animal model systems have been used to study the effects of cigarette smoke on development and physiology. These have helped to identify genetic and molecular mechanisms underlying issues of addiction and the wide variety of complications that arise from smoking both during development and in the adult [4, 12–14]. In recent years, human embryonic stem cells (hESCs) have been used to study the effects of smoking on developmental processes including pluripotency and differentiation [14, 15].

The current study builds on these important findings that have set the groundwork for comparative analysis of tobacco cigarette smoke and e-cigarette aerosol on developmental events. Despite the advance of protocols for directed differentiation of hESCs into definitive cell types, there have been no studies to our knowledge that have employed these protocols to provide focused insights into the impact of cigarette smoke on cardiac development. Here we utilize a high efficiency directed cardiac differentiation method as a disease modeling platform to address unanswered questions about the impact of tobacco smoke and e-cigarette aerosol on cardiac lineage specification. This approach is coupled with studies in zebrafish, a well-known medium-throughput animal model for heart development and toxicology studies [16–21], to assess toxicity as well as functional and morphological defects caused by exposure to cigarette smoke during development *in vivo*. The rationale for these studies was to determine the impact of tobacco smoke and e-cigarettes on cardiac development.

## Materials and Methods

### Smoke and Aerosol Extracts

Smoke and aerosol extracts were generated with an approach adapted from previous publications [6, 22]. To generate smoke extracts, 85 mL of DMEM-F12 medium was placed in a gas washing bottle. A vacuum was used to draw tobacco smoke or e-cigarette aerosol into the media through a gas diffuser to generate media containing the full contents of either 1 aerosolized e-cigarette cartridge (South Beach Smoke, Tobacco Classic, Full Flavored, 16 mg nicotine/cartridge) or the smoke generated from 22 tobacco cigarettes (University of Kentucky, 3R4F Research grade cigarettes). Given that e-cigarettes can have a wide range of effects on cell physiology [13], we opted to study a single common brand e-cigarette in greater detail rather than a sampling of many types in lesser detail. Tobacco cigarettes were purchased from the University of Kentucky. These tobacco cigarettes are fully defined and are established for research grade studies (http://www2.ca.uky.edu/refcig/). Purified nicotine (Sigma) in media was used as a control. Tobacco cigarette extracts, e-cigarette aerosol extracts, and purified nicotine samples were aliquoted into 1 mL volume and frozen at -80C. To quantify the nicotine concentration, all samples were analyzed using gas chromatography (GC) by the UCSF Clinical Pharmacology Laboratory. Concentrations of nicotine were determined by gas chromatography with nitrogen-phosphorus detection [23], using 5-methylnicotine as an internal standard. This method has been modified for simultaneous extraction of nicotine and cotinine with determination using capillary GC [24]. The limits of quantitation are 1 ng/ml for nicotine.

### Cigarette extract exposure

Cleavage stage zebrafish (*Danio rerio*) embryos were continuously exposed for 3 days in non-tissue culture plates (1 embryo per 0.5 mL) containing purified nicotine, e-cigarette or tobacco extracts (6.8, 13.7 and 34 μM nicotine) in fish water containing 0.1% dimethyl sulfoxide (DMSO; sigma) or vehicle control (0.1% DMSO). DMSO was used to increase permeability of the chorion [25] and exposures were replenished every 24 h. Cigarette smoke extracts were diluted in either culture medium or fish water and nicotine concentration was used as a common reference point. For *in vitro* assays, cells were treated with varying doses (1.7, 3.4, 6.8 or 13.7 μM) of nicotine from tobacco smoke extract, e-cigarette aerosol extracts, or media containing purified nicotine diluted into culture medium. Extracts were added from the onset of differentiation (day 0) and added fresh at every media change. Control for in vitro differentiation assays was culture media alone.

### Zebrafish husbandry and assays

Wild-type (AB; Zebrafish International Resource Center, Eugene, OR, USA) zebrafish were bred and embryos were raised following procedures previously described [26]. Adult zebrafish were housed in 10 liter (L) aquaria at a density of ~5 fish per 1L with a 14 h/10 h light/dark cycle. Fish were fed Zeigler Adult Zebrafish Diet (Pentair Aquatic Eco-Systems) twice daily and recordings of water temperature (~27.5°C), pH (7.5), conductivity (800 μS) were collected daily. Single embryos were cultured in individual wells of multiwall plates to permit individual dosing and phenotyping. To assess vitality and growth following extract exposures: survival, hatching from chorion and pigment formation (Full, partial or none) were assessed every 24 h. At approximately 72 hours post exposure (hpe), incidence and severity of heart malformation was scored. Heart rate was determined by counting ventricular contractions over a period of one minute from randomly selected zebrafish larvae at 27°C. For qRT-PCR, zebrafish embryos (cleavage stage) were exposed to either control, e-cigarette or tobacco extracts at 13.7 μM and embryos were collected at 24 hpe for RNA isolation as described below. Bright field images were obtained with a Nikon SMZ1000 microscope using a Canon Rebel T3i camera. All experimental procedures involving animals were approved by the Institutional Animal Care and Use Committee at the University of Washington, Seattle. All assays consist of a minimum of three independent breeding trials and data were collected in a blinded fashion.

### Human ESC Directed Differentiation

Undifferentiated RUES2 hESCs (Female line, Rockefeller University, NIH registry number 0013) were plated at 1.6x10^5^ cells/cm^2^ on Matrigel (BD) coated plates and maintained in an undifferentiated state with mouse embryonic fibroblast (MEF) conditioned media containing 5 ng/mL hbFGF (Peprotech, 100-18B). Directed differentiations using a monolayer platform were performed based on previous reports [27] with a modified protocol. Undifferentiated hESCs were plated as single cells as described previously and upon reaching appropriate confluency, treated with the Wnt/β-catenin agonist CHIR-99021 (1 μM, Cayman chemical, 13122) for 24 hours. Cells were then exposed to Activin A (R&D SYSTEMS, 338-AC-050) (100 ng/mL) in RPMI/B27 medium (day 0). After 17 hours, media was changed to RPMI/B27 medium containing BMP4 (R&D SYSTEMS, 314-BP-050) (5 ng/mL) and CHIR-99021 (1 μM, Cayman chemical,13122). On day 3, media was changed to RPMI/B27 medium containing the Wnt/β-catenin antagonist XAV-939 (1 μM; Tocris, 3748). Media was then changed on day 5 to RPMI/B27 medium. From day 0 to day 5, the B27 supplement utilized did not contain insulin (Invitrogen, 0050129SA). From day 7–14 a B27 supplement with insulin was used (Invitrogen, 17504044). For assays assessing the onset and rate of beating, cultures were analyzed independently during differentiation, with each well counted as n = 1.

### qRT-PCR

For quantitative RT-PCR, total RNA was isolated using the RNeasy Miniprep kit (Qiagen). RNA quality and amount was determined using a Nanodrop spectrophotometer. First-strand cDNA was synthesized was performed using the Superscript III enzyme kit (Invitrogen). Quantitative RT-PCR was performed using Sensimix SYBR PCR kit (Bioline) on a 7900HT Fast-Real-Time PCR System (Applied Biosystems). For in vitro assays, the copy number for each transcript is expressed relative to that of HPRT. For in vivo assays, the copy number for each transcript is expressed relative to that of β-actin. Primers used for quantitative PCR are listed in S1 Table.

### Flow Cytometry

WT RUES2 differentiated cells were labeled for flow cytometry using the following antibodies: cardiac troponin T (Thermo Scientific, Ab-1 (13–11)) or smooth muscle actin (Abcam, Ab 32575) or corresponding isotype controls. Cells were analyzed using a BD FACSCANTO II (Beckton Dickinson, San Jose, CA) with FACSDiva software (BD Biosciences). Instrument settings were adjusted to avoid spectral overlap. Data analysis was performed using FlowJo (Tree Star, Ashland, Oregon).

### Immunofluorescence

Cells were prepared for immunofluorescence exactly as has been described previously for staining of hESC-derived cells [27]. In brief, cells were fixed with either 4% paraformaldehyde or methanol, permeabilized in PBS containing 0.025% Triton-X, and blocked in PBS containing 1.5% normal goat serum. Cells were stained with NKX2.5 (R&D Systems, Cat.# AF2444, 1:400), Mouse monoclonal anti-PAN cadherin (Sigma C1821, 1:500), mouse monoclonal anti cardiac troponin T (Thermo Scientific MS-295-P1, 1:400), monoclonal mouse anti-human Smooth Muscle Actin (DAKO, Clone 1A4, 1:500), and mouse monoclonal anti-alpha-Actinin (Sigma, A7732 Clone EA-53, 1:800) followed by secondary staining with AlexaFlour-594 Donkey Anti-Goat (Invitrogen lot #1180089, 1:200) or AlexaFlour-594 Goat Anti-Mouse (Invitrogen lot # 1219862, 1:200). Nuclei were counterstained with DAPI. For quantification of immunohistochemistry results, images were analyzed using ImageJ to quantify the pixel intensity of various proteins. NKX2-5 was normalized to DAPI and all other samples were normalized to phalloidin. Sarcomere length measurements were accomplished by measuring the distance between ten sarcomeres in α-actinin stained samples and that distance divided by ten to determine the length between each sarcomere. Measurements were generated from at least 3 different biological replicates with 40–90 sarcomeres measured per treatment group.

### Cell Stress Array

Protein samples were isolated from control, e-cigarette aerosol extract (6.8 μM), or tobacco cigarette smoke extract (6.8 μM) treated samples on day 14 of differentiation using RIPA buffer (Sigma). Samples were quantified for protein concentration by BCA protein assay analysis (Thermo Scientific, 23227). A total of 300 μg of protein (100 μg of protein from each of 3 biological replicates) was used to analyze abundance of cell stress proteins in each condition using the Proteome Profiler, Human cell stress array kit (R&D, ARY018).

### Statistics

Single variable analysis between 2 samples was compared by Student’s t-test. Single and multivariable assays were analyzed by one or two way ANOVA. Results are presented as mean ± SEM. For all results: * P < 0.05. All data were derived from at least six independent experiments (biological replicates). Each figure legend describes the number of biological replicates (n) used to generate statistical comparisons within each experiment.

## Results

This study was designed to determine the impact of tobacco cigarette smoke extract and e-cigarette aerosol extracts on cardiac development *in vivo* and *in vitro*. As opposed to using percent refill solution[13], puff equivalents (PE)[14] or concentrations of cigarette smoke condensate (CSC)[28] as used in a wide range of toxicological studies, we chose to normalize our groups to nicotine concentration, since nicotine is the primary determinant of smoking behavior and provides us with a quantifiable baseline with which to compare our results to other studies using a similar metric. Although supraphysiological with reference to doses measured in fetal serum [29], these doses were established within the working range of previous studies using embryonic stem cells and zebrafish for toxicology studies of nicotine (1.2–10 μM)[6, 15, 16]. Further discussion of this dosing rationale is provided below.

### The effects of cigarette exposure on zebrafish development

Zebrafish are an established *in vivo* medium-throughput vertebrate model for studying chemical toxicity and heart development [18, 19, 21, 30]. We used this system to explore the effect of cigarette exposure during *in vivo* development. To assess the effects of tobacco smoke and e-cigarette aerosol extracts on vertebrate development, zebrafish were reared for 72 h in cigarette extracts at 6.8, 13.7 and 34 μM nicotine. As indicators of general growth and heart development we collected data on survival, hatching from the chorion, pigment formation, incidence and severity of heart malformation and heart rate as described in the methods.

**Fig 1:**
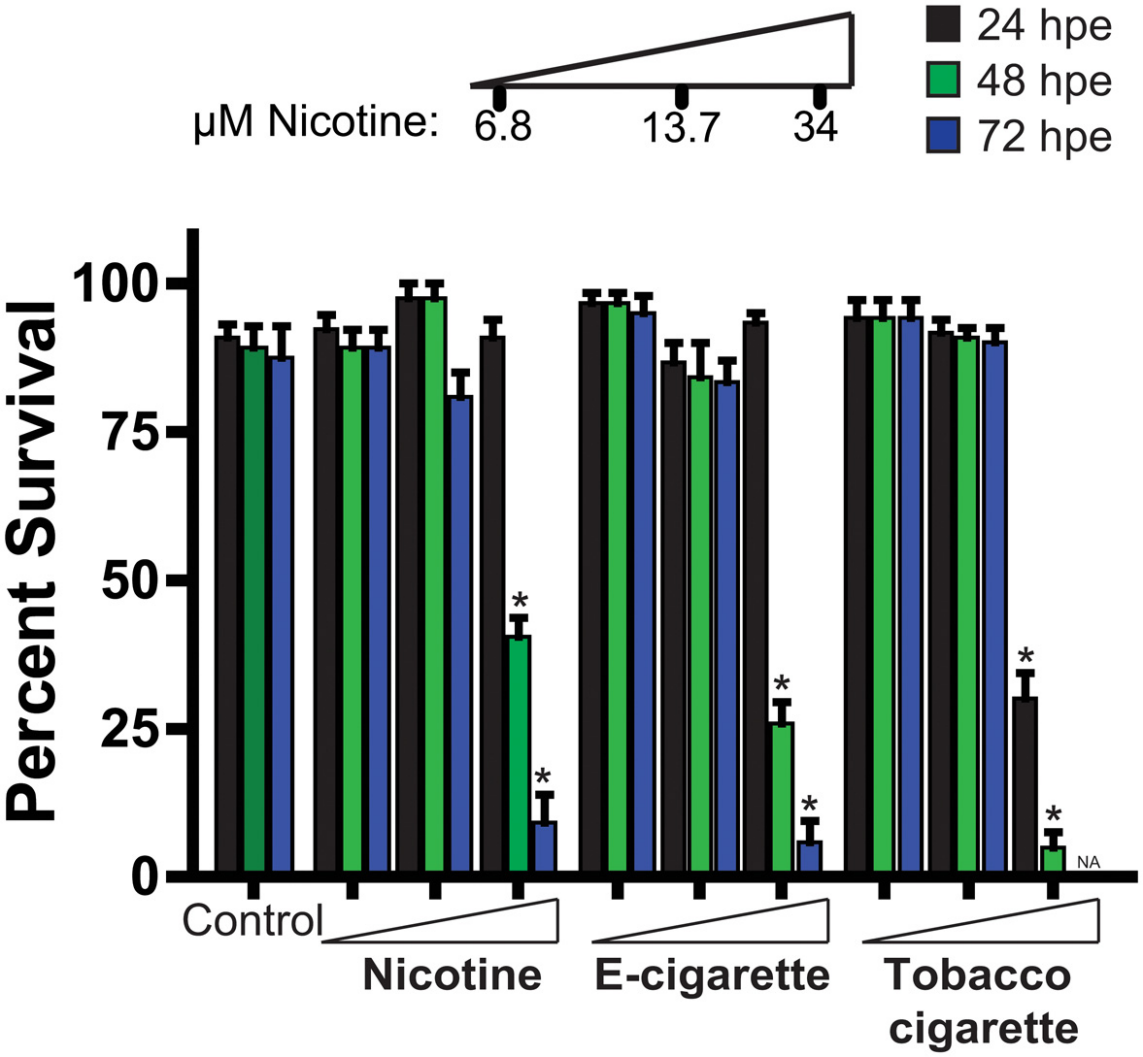
Zebrafish embryo-larval survival analysis following escalating doses of nicotine, e-cigarette and tobacco extracts. Assessment of zebrafish survival over 72 h with increasing concentrations of pure nicotine, e-cigarettes and tobacco cigarettes extracts or vehicle control (0.1% DMSO). n ≥ 3 (independent experiments with each n containing between 24–48 animals per treatment). * P < 0.05, hpe = hours post exposure.

Treatment with nicotine, e-cigarette aerosol extract, and tobacco smoke extract showed no change in embryo survival over the initial 24 hours at all the doses examined, with the exception of reduced survival observed in the 34 μM tobacco smoke extract treated cohort (Fig 1). Exposure to 34 μM nicotine, e-cigarette aerosol extract, and tobacco-cigarette smoke extract at 48 hrs resulted in markedly reduced survival when compared to controls. Although the 34 μM tobacco-exposed cohort had 0% survival by 72 hpe, exposure to the same dose with nicotine and e-cigarettes also had a significant impact in survival (nicotine: 9.2%, e-cigarette: 5.8%). (Fig 1). E-cigarette extract exposed zebrafish showed no striking differences in pigment formation and chorion hatching when exposed at 6.8 or 13.7 μM nicotine when compared to control (S1 Fig). Although 6.8 μM tobacco cigarette-exposed fish were similar to controls, decreased hatching and pigment formation was observed at the 13.7 μM nicotine (S1 Fig).

**Fig 2:**
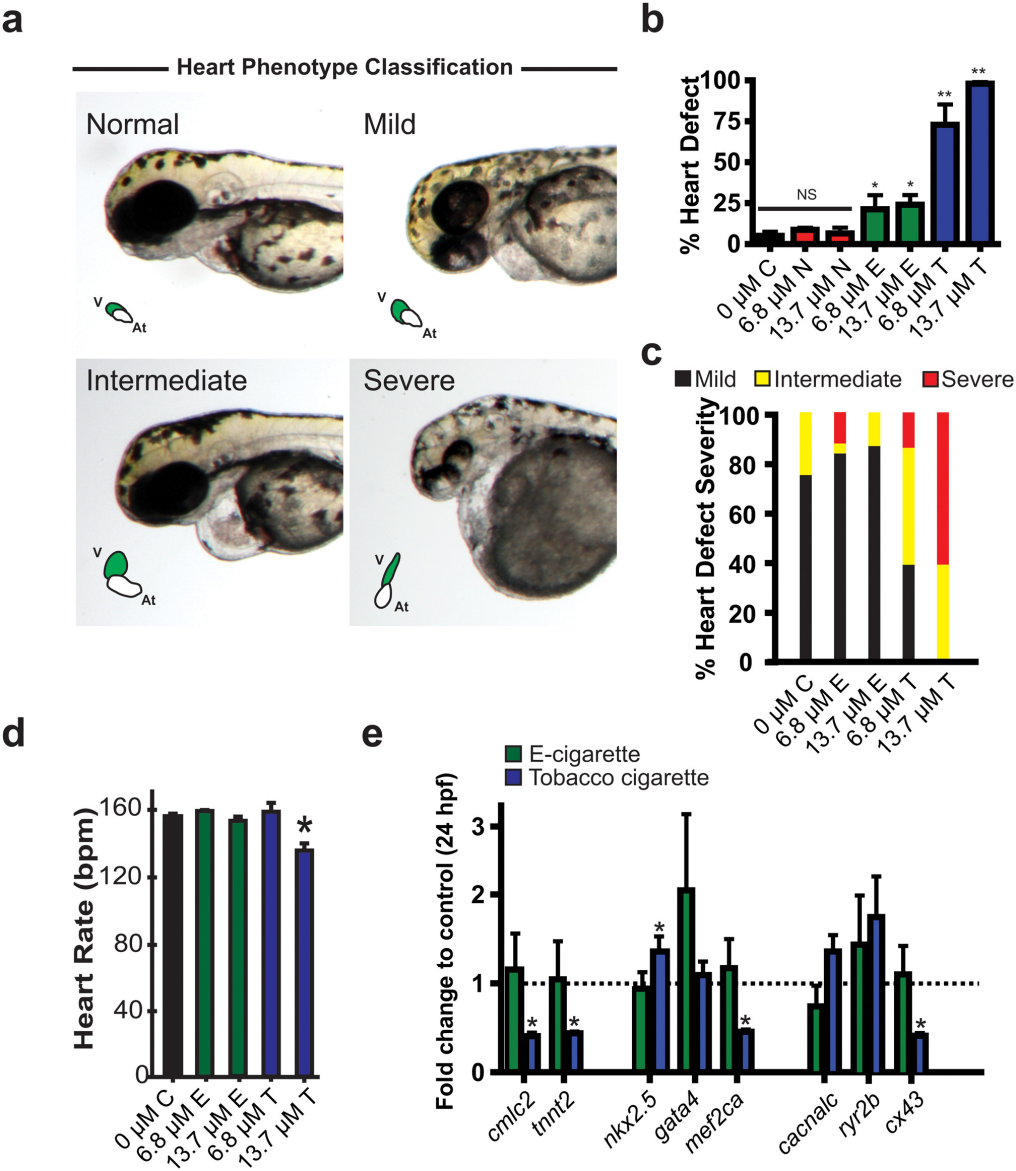
Cardiac developmental defects observed in zebrafish treated with cigarette smoke. (a) Representative whole mount images of zebrafish at 72 hpe showing normal, mild, intermediate, and severe cardiac developmental defects. v = ventricle, At = atrium. (b-c) Analysis of percent zebrafish with heart defects (b) severity of heart defects and (c). (d) Analysis of heart function in control, e-cigarette and tobacco treated groups at 72 hpe. (e) Quantitative RT-PCR analysis (fold change from control) of a panel of genes with critical roles in early heart development at 24 hpe. n ≥ 3 (independent experiments with each n containing between 24–48 animals per treatment). For qRT-PCR, n = 3 with each n consisting of 28–35 embryos from independent breeding pools. * P < 0.05, hpe = hours post exposure; N = Nicotine, E = E-cigarette, T = Tobacco.

To assay the effects on cardiac development, fish were scored for the incidence of heart abnormalities and their severity at 72 hpe. Four phenotypes were observed and were classified as: *normal*, looped heart with no pericardial edema [31]; *mild*, slight pericardial edema with looped heart; *intermediate*, unlooped, balloon shaped heart chambers coupled with pericardial edema; *severe*, stretched unlooped heart, no directional blood flow with an unabsorbed yolk (Fig 2a). Due to the overt lethality observed at 34 μM nicotine across all groups we assessed cardiac defects in the 0, 6.8 and 13.7 μM treatment groups. Analysis of zebrafish exposed to nicotine alone showed no significant difference from controls regarding the frequency of heart defects (Fig 2b). However, fish exposed to e-cigarette aerosol extract or tobacco cigarette extract showed markedly increased heart defect incidence with tobacco cigarette treated cohorts showing the most number and greatest severity of defects in a dose dependent manner (Fig 2b and 2c). To investigate heart function we quantified heart beating rate at 72 hpe. While 13.7 μM nicotine from tobacco cigarette exposed fish showed markedly decreased heart rate, e-cigarette aerosol extract exposure at an equivalent dose was not different from controls (control: 155 ± 1.7; e-cigarette: 152 ± 1.8; tobacco cigarette: 134 ± 11 bpm) (Fig 2d).

We also performed transcriptional profiling of 1 day old zebrafish embryos following a 24 h exposure to e-cigarette and tobacco extracts (13.7 uM). These data show a marked decrease in contractile proteins *cmlc2* and *tnnt2*, the transcription factor *mef2ca*, and the junctional protein responsible for electromechanical conduction in the heart (*cx43*) and a significant increase in the cardiac homeobox gene *nkx2*.*5* only in tobacco cigarette extract treated fish (Fig 2e). These data indicate that both e-cigarette aerosol extract and tobacco cigarette smoke extract exposure affects heart development and function with a more severe impact in the context of tobacco cigarette extract.

### Analysis of smoke exposure during cardiac directed differentiation of hESCs

**Fig 3:**
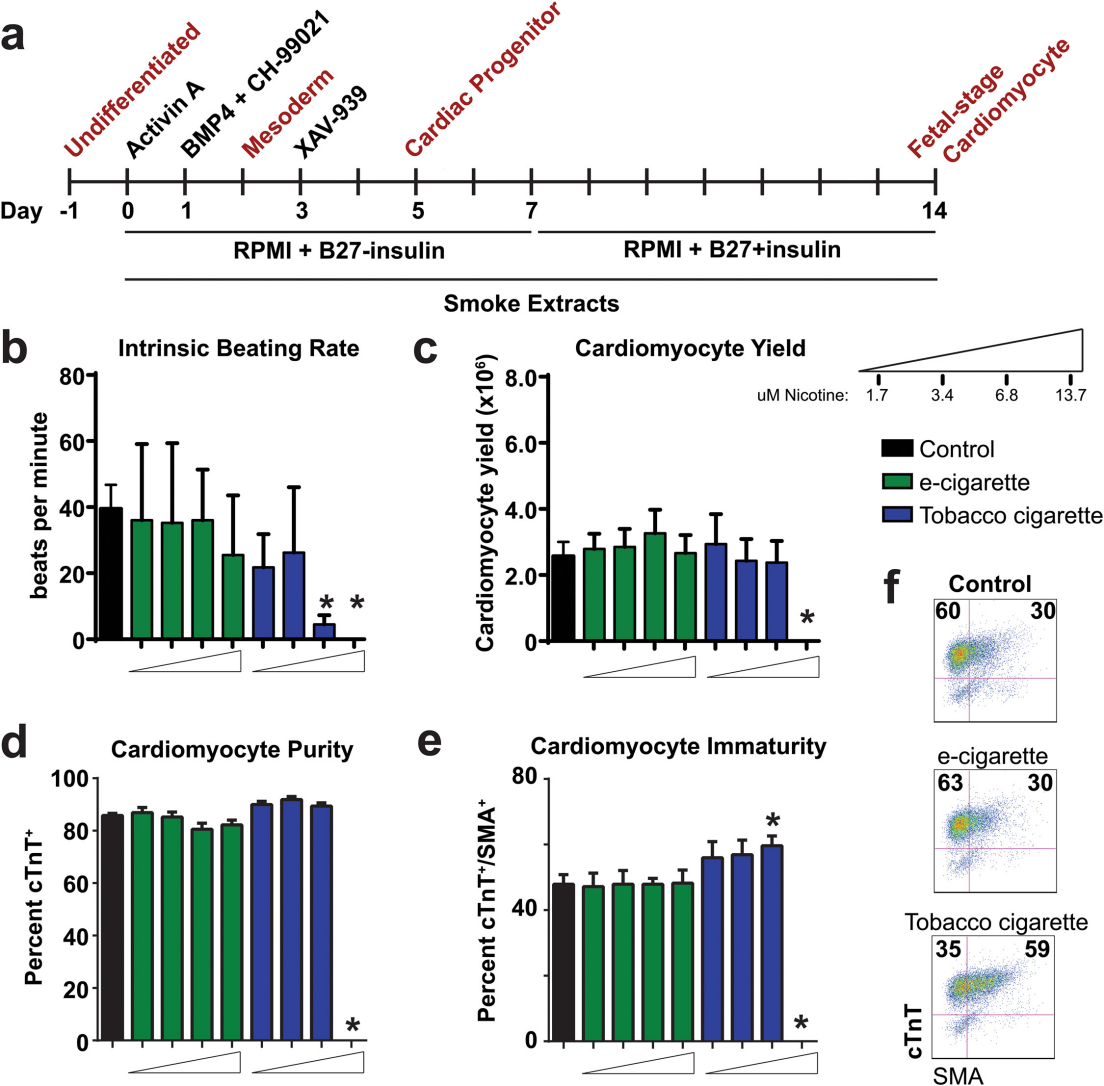
Analysis of e-cigarette and tobacco cigarette on hESC cardiac differentiation. (a) Timeline of differentiation protocol for cardiac directed differentiation of hESCs. (b-e) Analysis of cardiac endpoints including intrinsic beating rate (b) cardiomyocyte yield (c), cardiomyocyte purity (d), and cardiomyocyte immaturity based on percent cTnT^+^/SMA^+^ (e) and representative flow cytometry plots (f) on day 14 of differentiation with increasing doses of purified e-cigarette and tobacco cigarette extracts. n ≥ 6 per group. * P < 0.05.

To study the effects of smoke exposure on hESC cardiac development, we used a monolayer-based directed hESC cardiac differentiation platform [27, 32, 33] (Fig 3a). The serial induction of differentiation with Activin A and BMP4 signaling, in combination with the small molecule Wnt/β-catenin agonist CHIR-99021 leads to a transition from pluripotency (day 0) to lateral plate mesoderm (day 2). Use of the small molecule Wnt/β-catenin inhibitor XAV-939 at differentiation day 3 facilitates the transition from mesoderm to cardiac progenitors at day 5 of differentiation. Cells then progress to definitive fetal-stage cardiomyocytes by completion of the protocol (day 14). We utilized this protocol to assess the effects of smoke extracts on human cardiomyocyte differentiation (smoke extracts was added fresh at each media change througout the experimental time course) (Fig 3a).

In an initial dose-escalation analysis, we compared control cohorts to those exposed to e-cigarette aerosol extractor tobacco smoke extract assayed at day 14 of differentiation. During the time course of differentiation, we first observed that tobacco cigarette smoke extract at a dose of 13.7 μM nicotine was cytotoxic, resulting in cell death within 2 days of differentiation (Fig 3b–3e). Among all other viable samples, we carried out a general assessment of cardiomyocyte development at day 14 on the basis of intrinsic beating rate (beats/minute), cardiomyocyte yield (total number of cardiomyocytes harvested at day 14), cardiomyocyte purity (cTnT^+^ cells at day 14), and measures of cardiomyocyte immaturity (total cells double positive for cTnT and SMA). Compared to control samples which had an intrinsic beating rate of 39.5 ± 7.2 beats per minute, we found no difference in the beating rate of samples treated with e-cigarette aerosol extract. However, tobacco cigarette smoke extract treatment at the 6.8 μM nicotine dose showed significant reduction in intrinsic beating rate (4.5 ± 1 beats per minute) (Fig 3b). Analysis of cardiomyocyte yield and purity (based on cTnT^+^ cells) showed no difference between groups with the exception of the highest dose of tobacco cigarette extract (Fig 3c and 3d). Smooth muscle actin (SMA) is known to be expressed early in cardiac development but is progressively reduced as cells mature[34]. We therefore assessed cardiac immaturity on the basis of cells co-expressing cTnT and SMA at day 14 and found that tobacco cigarettes showed increased percent double-positive cells at the 6.8 μM nicotine dose (Fig 3e and 3f). We found that cells differentiated in purified nicotine were not significantly different on the basis of all these endpoints compared to control samples (S2 Fig). These initial studies illustrated the cytotoxic effect of tobacco cigarette extract exposure on developing hESCs and revealed its inhibitory effect on cardiomyocyte differentiation.

### Analysis of early cell state transitions during hESC cardiac directed differentiation

Given results from initial studies looking at increasing doses of nicotine from different cigarette sources, a dose of 6.8 μM nicotine was chosen to compare the effects of e-cigarette aerosol extract and tobacco cigarette smoke extract to control samples during a time course analysis. To determine the impact of cigarette smoke treatment on different stages of cardiac differentiation, RNA samples were harvested at day 2 (mesoderm), day 5 (cardiac progenitor cell), and day 14 (definitive fetal-stage cardiomyocytes). Quantitative RT-PCR analyses were performed to determine the transcript abundance of a panel of genes known to have critical roles in cell fate decisions or to participate in the functional development of the cardiomyocyte at each of these stages.

**Fig 4:**
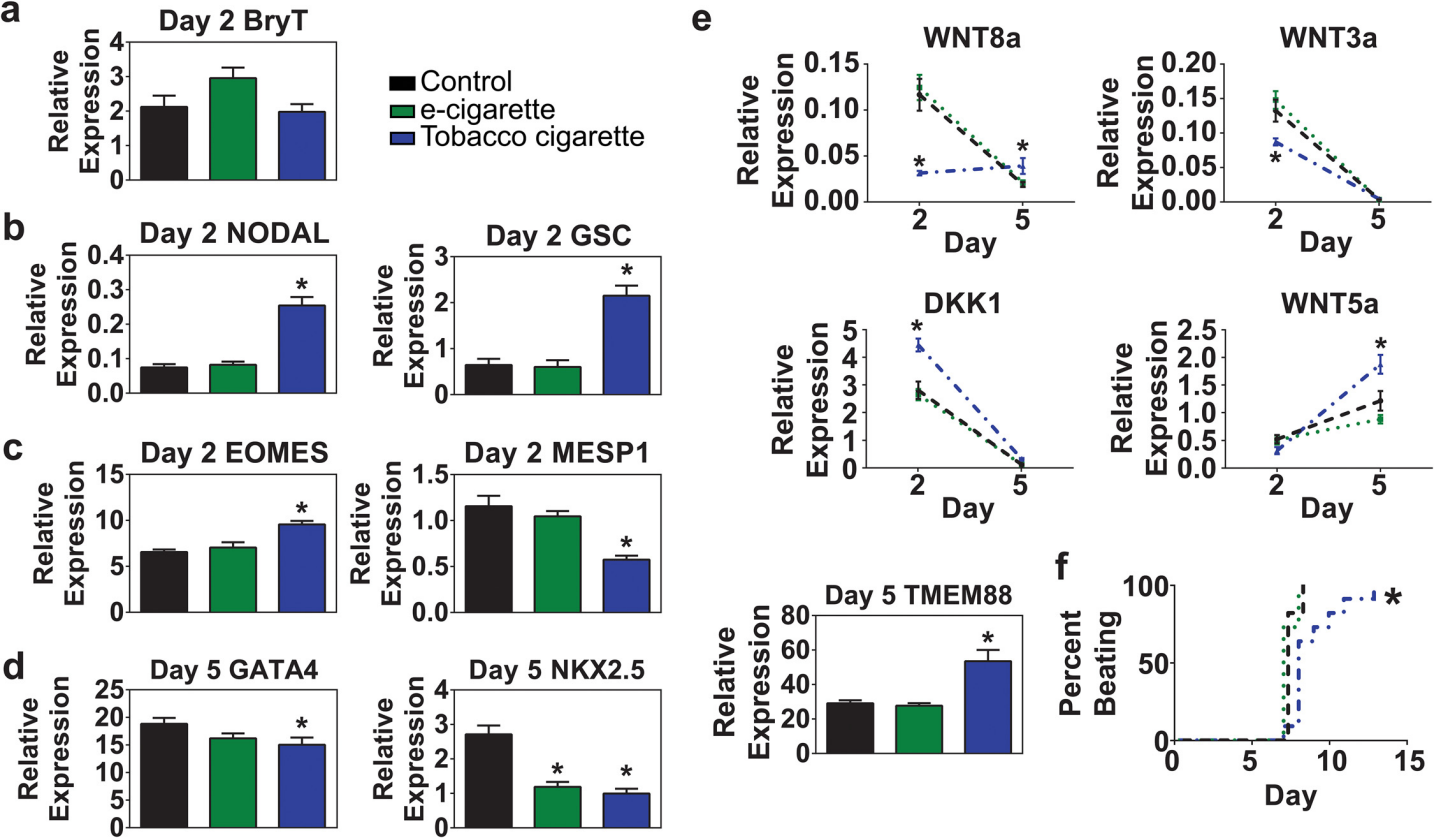
Impact of cigarette exposure on cardiogenic mesoderm development. (a-c) Quantitative RT-PCR analysis of expression levels on day 2 of differentiation for genes involved in mesoderm development following exposure to 6.8 μM e-cigarette, or tobacco cigarette extract. Analysis included the pan mesendoderm marker Brachyury T (T) (b), mesendoderm genes involved in patterning anterior primitive streak, NODAL and goosecoid (GSC) (c), and cardiogenic mesoderm genes eomesodermin (EOMES) and MESP1. (d) Quantitative RT-PCR analysis of cardiac progenitor cell markers on day 5 of differentiation including GATA4 and NKX2.5. (e) Quantitative RT-PCR analysis of Wnt/β-catenin ligands WNT3a, WNT8a, WNT5a and Wnt/β-catenin signaling inhibitors DKK1 and TMEM88 between days 2 and 5 of differentiation. (f) Time course analysis of onset of beating during cardiac differentiation. n ≥ 6 per group. * P < 0.05.

During the transition through mesoderm on day 2, we assessed expression of the pan mesendoderm marker Brachyury T and found no difference between control, e-cigarette aerosol extract and tobacco cigarette smoke extract treated samples (Fig 4a). However, genes involved in patterning anterior primitive streak-derived mesendoderm development including the bicoid homeobox protein Goosecoid (GSC) and NODAL were significantly higher only in cells treated with tobacco cigarette smoke extract (Fig 4b). Among the transcription factors known to specify the early stages of cardiac development, eomesodermin (EOMES) is known to regulate MESP1 in an axis of signaling to directed pre-cardiac mesoderm fate specification [35]. We found that cells treated with tobacco cigarette smoke extract had significantly higher levels of EOMES and lower levels of MESP1 compared to control and e-cigarette aerosol extract treated samples (Fig 4c). We also analyzed a panel of genes involved in transition through the cardiac progenitor cell stage (day 5). GATA4 and NKX2.5 are canonical cardiac transcription factors known to mediate expression of a broad range of cardiac developmental and structural genes [36, 37]. Both e-cigarette aerosol extract and tobacco smoke extract treated samples showed significantly reduced levels of NKX2.5 compared to control samples. Tobacco smoke extract treated samples also showed significantly lower expression of GATA4 (Fig 4d).

Wnt/β-catenin has long been known to be a critical mediator of cardiac development where stimulation of the pathway is required for transitioning through mesoderm and down-regulation of Wnts are required to mediate specification into the cardiac lineage [27, 38]. Expression analyses of Wnt modulators found that samples treated with tobacco cigarette smoke extract had significantly lower expression of canonical Wnt ligands WNT3a and WNT8a as the cells transitioned through mesoderm on day 2 of differentiation (Fig 4e). Expression of both ligands was down-regulated for most samples during the transition from mesoderm (day 2) to cardiac progenitor (day 5) stage with the exception of WNT8a which was sustained in the tobacco cigarette group. In contrast, the non-canonical ligand WNT5a (β-catenin-independent) was up-regulated in all groups during the transition to the cardiac progenitor cell, however, a significant difference in expression levels was observed between the tobacco treated samples over that observed with control and e-cigarette aerosol extract treated samples (Fig 4e). Tobacco smoke extract treated samples showed significantly higher levels of DKK1 on day 2 of differentiation with down-regulation of this WNT inhibitor observed in all groups by day 5. Lastly, we analyzed TMEM88, a transmembrane protein known to inhibit Wnt/β-catenin signaling by binding to Disheveled [27, 39]. We found that TMEM88 was significantly up-regulated in tobacco extract treated samples. These data show significant dysregulation of a key signaling pathway required for fate specification in cardiac development following exposure to tobacco cigarette extracts.

### Analysis of cigarette exposure on fetal stage myocytes generated from hESCs

We assessed the onset of beating during the progression from cardiac progenitors at day 5 to definitive cardiomyocyte development at day 14 (Fig 4f). These data indicate that control and e-cigarette aerosol extract treated samples showed active contraction around day 7–8 of differentiation. However, tobacco smoke extract treated samples were significantly delayed in the onset of beating with contraction occurring variably between days 7 and 13 of differentiation (Fig 4f).

**Fig 5:**
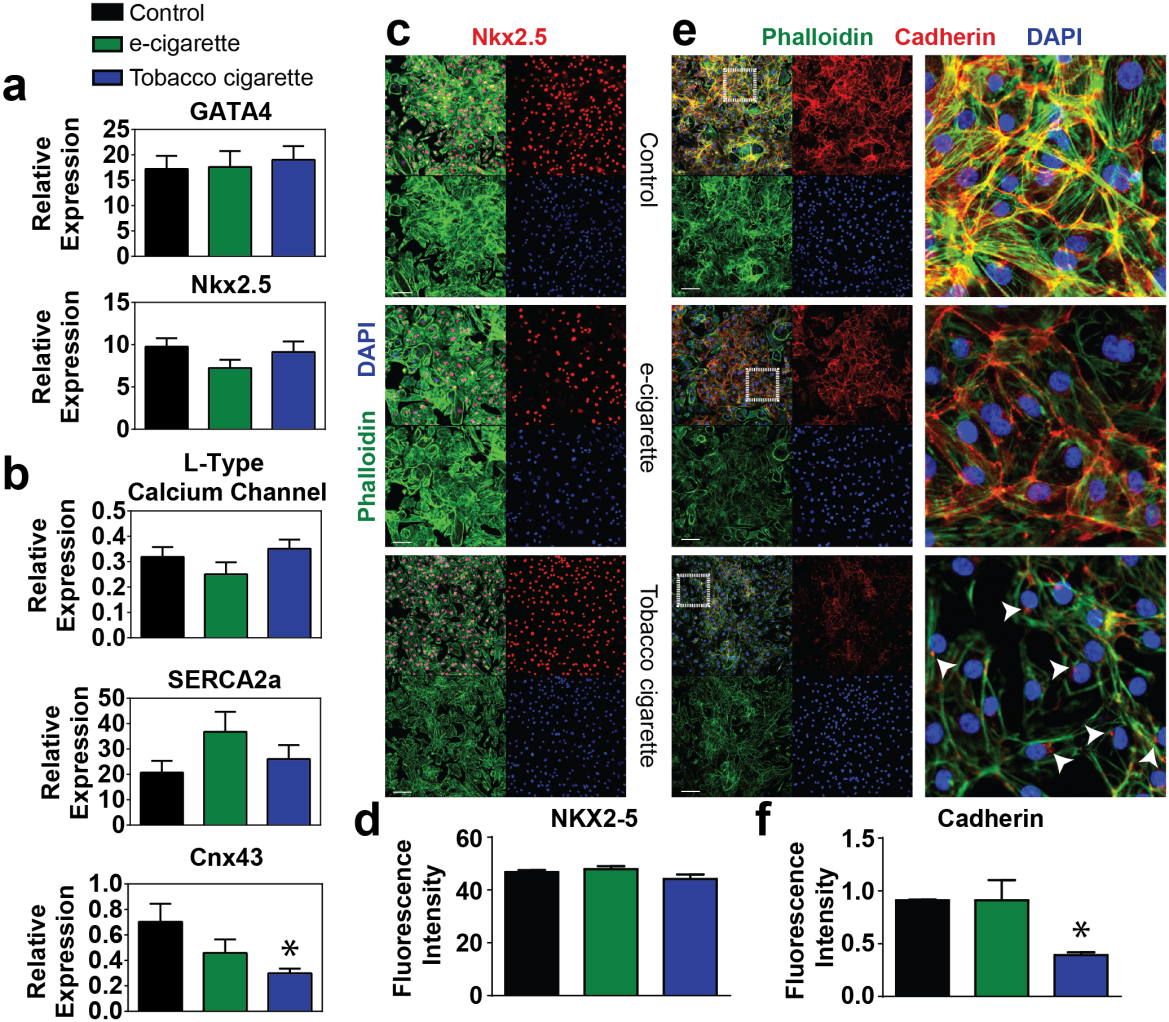
Analysis of hESC derived fetal cardiomyocyte transcription factor, calcium handling, and junction protein expression. (a) Expression level of cardiac transcription factors GATA4 and NKX2.5 (a) and calcium handling proteins including the L-type calcium channel and SERCA2a, and the junctional protein CNX43 (b) by quantitative RT-PCR in cells treated with 6.8 μM e-cigarette or tobacco cigarette extracts vs. control. (c-d) Representative immunocytochemistry (c) and quantification (e) for NKX2.5 in fetal cardiomyocytes with various cigarette treatments compared to control. (e-f) Representative immunohistochemistry (e) and quantification (f) for the junction protein cadherin in fetal hESC cardiomyocytes with various cigarette treatments compared to control. Inset shown to the right. Arrows indicate perinuclear expression of cadherin. n ≥ 6 per group. Scale bar = 100 μm. * P < 0.05.

Human ESC-derived cardiomyocyte samples were analyzed for a panel of transcription factors, calcium handling proteins, and contractile proteins that have been shown to have important roles in cardiac development and function. In contrast to day 5 samples which exhibited a reduction in cardiac transcription factors, we found that GATA4 and NKX2.5 mRNA levels were not different between groups at day 14 (Fig 5a). Immunohistochemical analysis of Nkx2.5 protein expression corroborated this finding (Fig 5c and 5d). Furthermore, analysis of calcium handling proteins such as the L-type calcium channel and SERCA2a showed no difference in expression between e-cigarette aerosol extract and tobacco smoke extract treated samples compared to control (Fig 5b). However, the junctional protein connexin 43 which is responsible for coordinating electromechanical transduction in cardiomyocytes was significantly down-regulated in tobacco extract treated samples compared to control or e-cigarette treated cohorts (Fig 5b).

We also assessed the junction protein cadherin involved in signaling and structural integrity during cardiac development. During embryogenesis cadherins accumulate in the Golgi apparatus and then shuttle to the membrane as cells mature. Immunohistochemical analysis showed uniform cadherin expression in control cardiomyocytes and e-cigarette aerosol extract treated samples with robust localization at the cell membrane and cell-cell junctions. In contrast, tobacco cigarette smoke extract treated samples had very low levels of cadherin protein expression that was localized in the peri-nuclear domain as opposed to the cell membrane (Fig 5e and 5f and S3 Fig).

**Fig 6:**
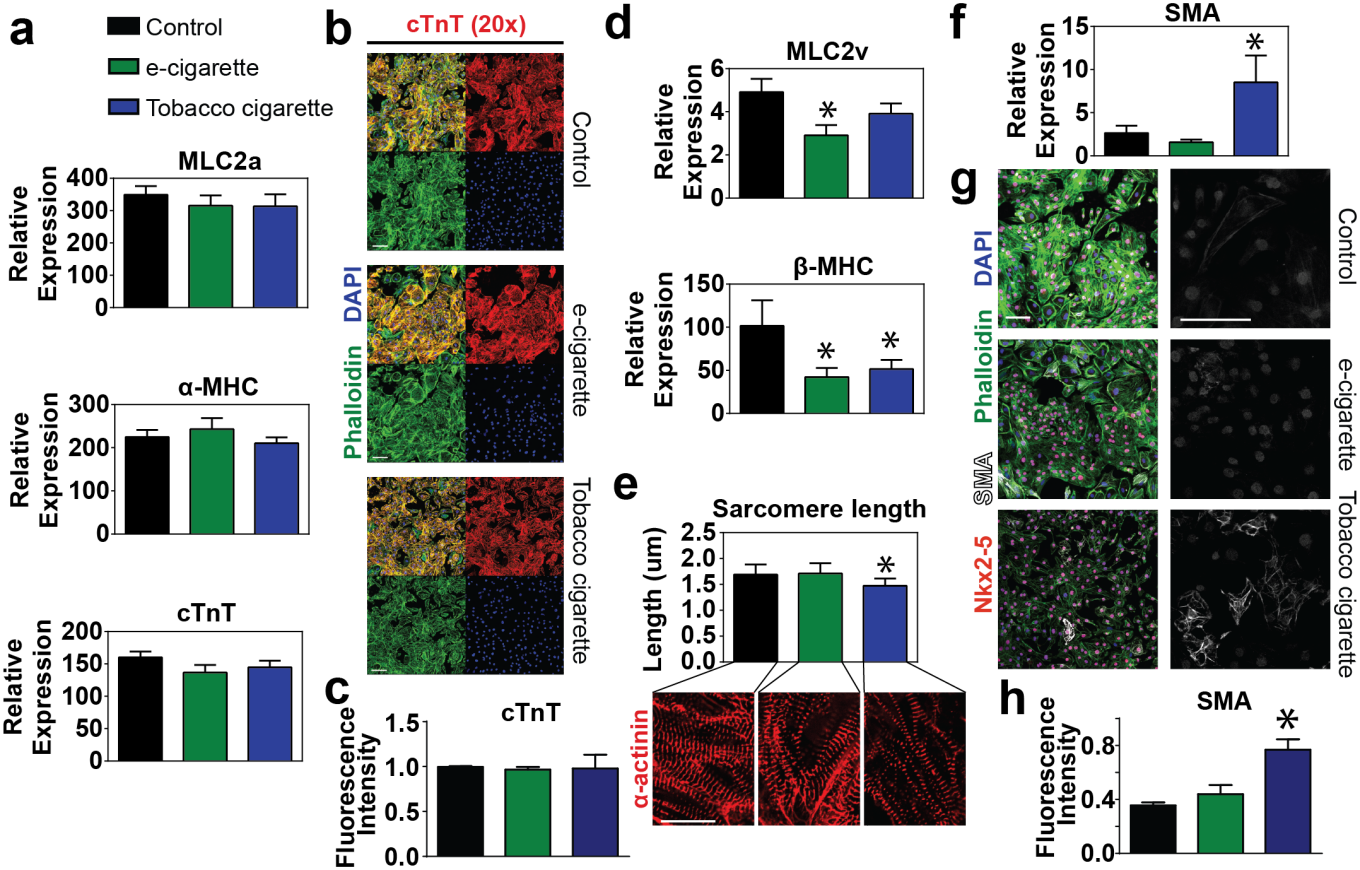
Analysis of cardiac myofilament and structural protein expression. (a) Quantitative RT-PCR analysis of early developmental myofilament proteins including the atrial myosin light chain MLC2a, the myosin isoform α-MHC and cardiac troponin T (cTnT) in cells treated with 6.8 μM e-cigarette or tobacco cigarette extracts vs. control. (b-c) Immunohistochemistry (b) and quantification (c) of the myofilament proteins cardiac troponin T (cTnT) in combination with phalloidin and nuclear counterstain DAPI in control cells or those treated with 6.8 μM e-cigarette or 6.8 μM tobacco cigarette extracts. Scale bar = 100 μm for cTnT. (d) Quantitative RT-PCR analysis of mature developmental myofilament isoforms including the ventricular myosin light chain MLC2v and the myosin isoform β-MHC in cells treated 6.8 μM e-cigarette or tobacco cigarette extracts vs. control. (e) Quantitation of sarcomere length as measured from samples stained for α-actinin by immunohistochemistry comparing control vs. 6.8 μM e-cigarette or tobacco cigarette. (f-h) Quantitative RT-PCR (f) immunohistochemistry (g) and quantification of IHC (h) for the immature cardiac marker smooth muscle actin (SMA) in control vs. cells treated with 6.8 μM e-cigarette or tobacco cigarette extract. n ≥ 6 per group. Scale bar = 100 μm. * P < 0.05.

Lastly, we analyzed the expression levels of a variety of contractile proteins involved at various stages of maturation during cardiomyocyte development. Early developmental myofilament genes including the atrial isoform of myosin light chain (MLC2a), the fast ATPase alpha isoform of myosin heavy chain (α-MHC/MYH6), and the thin filament protein cardiac troponin T (cTnT) were shown to be expressed equivalently in all groups (Fig 6a). These findings were confirmed by immunohistochemical analysis of day 14 cells demonstrating similar expression of cTnT between all groups (Fig 6b and 6c). During development isoform switching of myofilament proteins is known to occur reflecting changes in the maturation state[40]. In particular, myocyte development involves a switch from the atrial myosin light chain (MLC2a) to the ventricular specific myosin light chain (MLC2v) and similarly the early development myosin isoform (α-MHC) is replaced with the adult slow ATPase myosin isoform (β-MHC). Although all groups showed similar expression of the early developmental variants, both e-cigarette aerosol extract and tobacco cigarette smoke extract treated samples had significantly lower expression of mature developmental marker β-MHC. Additionally, e-cigarette aerosol extract treated samples exhibited significantly lower levels of MLC2v (Fig 6d).

Sarcomerogenesis was used as another marker of developmental maturity in which sarcomere length was measured between control and cigarette treated samples. We found that control and e-cigarette aerosol extract treated samples had myofilaments with longer, more mature sarcomere lengths (Control: 1.68 ± 0.03 μm; e-cigarette: 1.70 ± 0.03 μm) compared to tobacco cigarette extract treated samples which showed etiolated myofilaments with shorter sarcomere lengths (1.47 ± 0.02 μm) (Fig 6e). Lastly, we analyzed smooth muscle actin expression as a marker of myocyte immaturity. Much as we observed in flow cytometry outcomes from initial dosing assays (Fig 3e and 3f), we found that transcript levels analyzed by RT-PCR and protein abundance by IHC showed significantly higher SMA expression in tobacco extract treated samples compared to either control or e-cigarette aerosol extract treated samples (Fig 6f–6h). Taken together, these data indicate that tobacco cigarette smoke extract treated samples have significant developmental deficiencies with more modest defects observed in e-cigarette aerosol extract treated cohorts.

We also determined whether a broad-based cellular stress response was activated with cigarette smoke exposure. To address this question, we tested whether markers of stress-related signaling cascades were significantly up-regulated in hESC-derived cardiomyocytes treated with both types of cigarette extracts compared to control samples. Protein samples from day 14 fetal cardiomyocytes differentiated with continuous exposure to e-cigarette and tobacco extracts (6.8 μM) were isolated and profiled for 26 different stress related proteins including redox enzymes, oxidative stress proteins, heat shock proteins, and proteins involved in NFκB and p53 signaling pathways. These results show that exposure to smoke resulted in no significant differences in stress-related proteins between the tested conditions (S4 Fig).

## Discussion

It is well established that smoking impacts development, causing a wide range of pregnancy-related problems including effects on heart development and function[2–4]. With the growing popularity of electronic cigarettes, these studies establish an important basis for understanding the impact of e-cigarettes and tobacco cigarettes in various physiological contexts. Here we sought to understand the implications for e-cigarette and tobacco smoke extract on cardiac development using *in vitro* human embryonic stem cell cardiac differentiation as well as zebrafish development *in vivo*. Although there are species dependent differences in the sensitivity to tobacco related chemicals such as aromatic hydrocarbons, our findings show a strong correlation between studies in developing zebrafish and human hES cardiac differentiation. Overall, these findings showed little to no cardiac developmental deficiencies in the context of nicotine alone but significant impact of tobacco cigarette extracts and e-cigarette aerosol extracts on heart development with more severe defects observed in the context of tobacco cigarette extract.

One of the major advantages of the current study included the capacity to study stage specific impacts of cigarette extracts during cardiac differentiation from hESCs. Upon mesoderm induction (day 2) we found that tobacco cigarette extract treated samples showed increased levels of anterior primitive streak markers GSC and NODAL and decreased Wnt ligand expression. This is in keeping with findings by Liszewski et al [6]. Assessment of cardiogenic mesoderm markers showed that tobacco cigarette extract treated samples had increased expression of eomesodermin (EOMES) and decreased MESP1 with correlative dys-regulation of Wnt signaling molecules. Collectively these data suggest two potential outcomes of cardiogenic mesoderm specification defects in tobacco extract treated samples: either there is 1) a shift in mesoderm patterning during gastrulation toward a more anterior primitive streak phenotype or 2) a delay in development through lateral plate mesoderm in cells treated with tobacco cigarette smoke. Both interpretations would account for observed differences in gene expression.

As the cells progress to the cardiac progenitor cell state we observed decreased expression of cardiac transcription factors in both e-cigarette aerosol extract and tobacco cigarette smoke extract treated samples. This further validates a delay in differentiation for tobacco cigarette smoke extract treated samples and indicates that e-cigarette aerosol extracts directly impact key regulators of early cardiac specification.

Assessment of e-cigarette smoke treatment showed evidence of inefficient maturation based on increased incidence of heart defects in developing zebrafish and reduced expression of late markers of maturation during hESC cardiac differentiation including expression of MLC2v and β-MHC. In addition to dysregulation of gene expression, tobacco cigarette smoke extract exposure resulted in a wide spectrum of cardiac defects in developing zebrafish and, in the context of hESC cardiac differentiation, showed delayed onset of beating, perinuclear localization of the junctional protein cadherin, increased expression of smooth muscle actin, and etiolated myofilaments with reduced sarcomere length. A reduction in beating rate seen in zebrafish and hESC cardiac differentiation in the context of tobacco cigarette extract is in keeping with previous studies[41] and may result, in part, from lower levels of junctional proteins including cadherin and connexin 43 during cardiac development. Further analysis indicated that these deficiencies are not likely the consequence of a general activation of stress related pathways.

Dosing considerations are always important in toxicology studies. Few studies have directly measured concentrations of tobacco metabolites in the human fetus. The most definitive data of which we are aware come from Luck et al. [29], who reported that newborns of smoking mothers had serum nicotine values ranging from 0.5–25 ng/ml (3.1–154 nmoles/liter). (A point of confusion in this area is the study by [6], who appear to have miscalculated the molar concentrations of nicotine from the study of Luck et al.: 0.5–25 ng/ml[29] calculated as 0.3–15.4 μM[6] should be 0.003-.154 nM). In any case, toxicology studies of zebrafish, cell culture models and, more recently, human pluripotent stem cells, typically showed that chronic nanomolar to low micromolar doses of nicotine show no effect on physiological or cellular endpoints, despite well documented human developmental defects associated with nicotine exposure [2–4]. As a consequence, nicotine concentrations used in toxicology studies fall largely within the micromolar range [6, 15–17]. Part of this dosing discrepancy may be explained by the fact that fetal tissues concentrate nicotine levels above those measured in the serum. Additional studies of fetal nicotine in a contemporary human cohort and further discussion on the proper approach for designing (e.g. dosing, normalization to nicotine vs. cotinine etc.) and interpreting toxicology assays associated with nicotine would be valuable.

This study has built on the work of many other labs making use of developmental model systems to study the effects of tobacco smoke and e-cigarettes on cell physiology[4, 6, 12–15, 28, 42]. Taken together, our data using a high efficiency cardiac directed differentiation of hESCs *in vitro* and zebrafish development *in vivo* indicate a dose dependent cytotoxic effect of cigarette smoking. The collective picture indicates that cigarette smoke treatment primarily delays development from the onset of differentiation with continuous impacts on progression to cardiac progenitor cells and to the fetal cardiomyocyte cell stage. It is not surprising that exposure to tobacco cigarette extract resulted in a broader spectrum of cardiac abnormalities than exposure to e-cigarette aerosol extracts. However, this study revealed that exposure to e-cigarette aerosol extracts also results in detrimental effects on cardiac development even though they lack most of the 7,000+ chemicals contained in tobacco cigarette smoke extracts.

The finding that nicotine treatment alone recapitulated untreated controls indicates that the impact of e-cigarette and tobacco cigarette on heart development is the consequence of other components. Many chemicals, including known ingredients in tobacco cigarettes such as polycyclic aromatic hydrocarbons can cause gross morphological defects [43–46]. Reports have also shown significant toxicity from e-cigarette treatment [12, 13, 47] with some reports showing the cause to be the presence of cinnamaldehyde and 2-methoxycinamaldehyde in e-cigarette flavorings [48, 49], the presence of formaldehyde in e-cigarette vapor[50], and tin particles in cartomizer fluid [51]. Other studies have emerged supporting this observation and raising the awareness of the risks associated with e-cigarettes particularly for women who smoke or “vape” during pregnancy [12, 13, 42, 47–49, 51]. Further studies using hESC directed differentiation toward other cell types will broaden our understanding of the impact of cigarette smoke during human development.

## Supporting Information

S1 Fig Developmental landmarks including percent with pigment formation (b) and percent hatched (c) at 48 and 72 hpe with exposure to increasing doses of e-cigarettes or tobacco cigarettes. n = 48 fish per group. hpe = hours post exposure; E = E-cigarette, T = Tobacco.
(PNG)

S2 Fig Effects of nicotine on cardiac differentiation. Cells were differentiated into the cardiac lineage and assayed for different markers of cardiomyocyte development at day 14 including intrinsic beating rate (a), cardiomyocyte yield (b), cardiomyocyte purity based on percent cTnT+ cells (c), and cardiomyocyte immaturity based on co-expression of cTnT and SMA (d). (e) Representative flow plots.
(PNG)

S3 Fig Cadherin expression in definitive cardiomyocytes. Images are an expanded version of those shown in Fig 5d.
(PNG)

S4 Fig Cell stress analysis of fetal cardiomyocytes treated with cigarette extracts. (a) Raw array results used for quantification of 26 cell stress proteins in control, e-cigarette, and tobacco cigarette treated samples. (b) Coordinates for identification of proteins represented in (a). (c) Quantification of mean pixel intensity for each cell stress protein in e-cigarette and tobacco cigarette treated samples compared to control (dotted line).
(PNG)

S1 Table Human and zebrafish primer sequences for quantitative RT-PCR. (DOCX)
