# Supplementary figures and images for: Cardiac Development in Zebrafish and Human Embryonic Stem Cells Is Inhibited by Exposure to Tobacco Cigarettes and E-Cigarettes
Source: PLoS One. 2015 May 15;10(5):e0126259. doi: 10.1371/journal.pone.0126259 (PMC4433280; doi:10.1371/journal.pone.0126259)

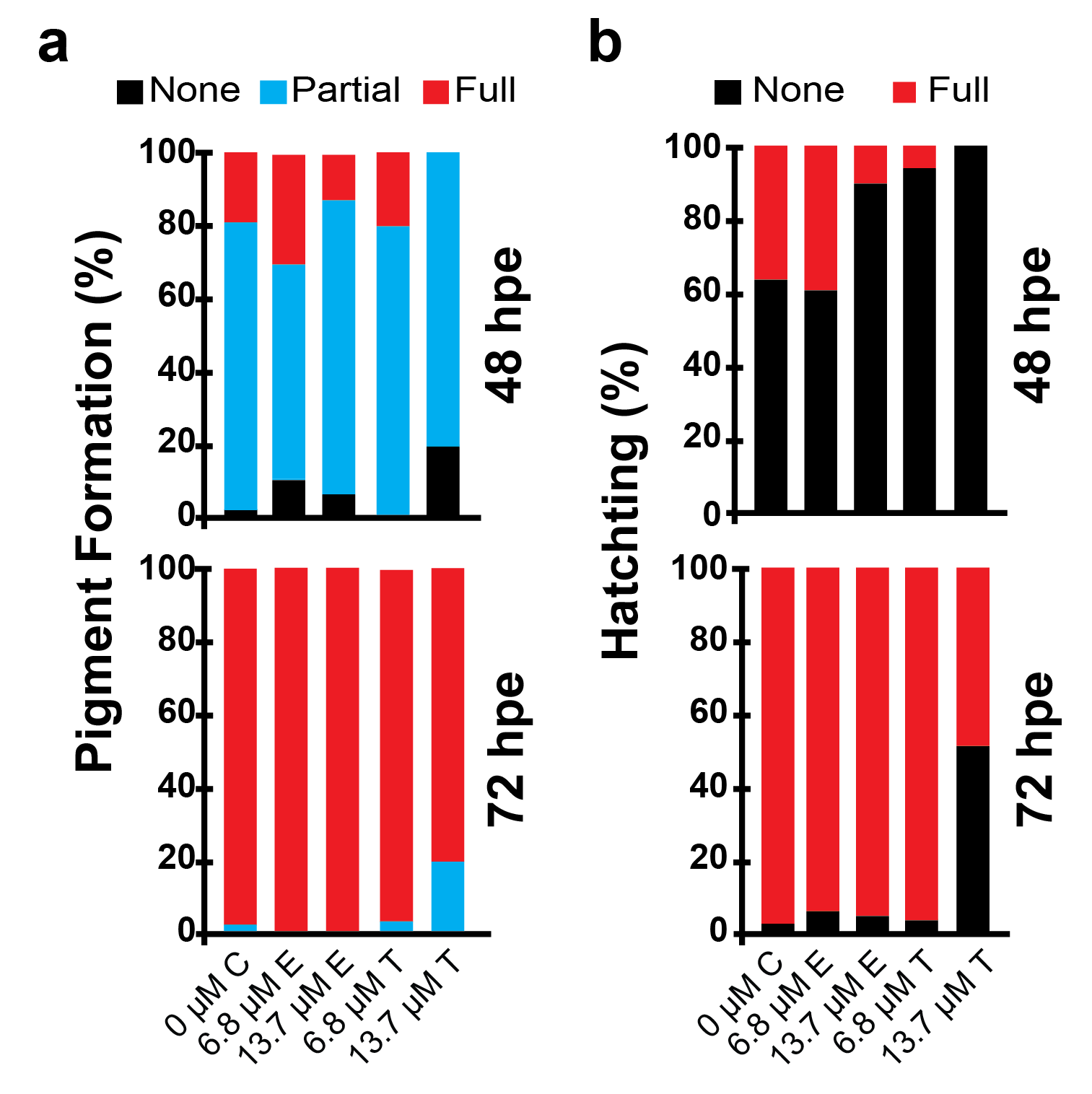

Supplement: S1 Fig — n = 48 fish per group. hpe = hours post exposure; E = E-cigarette, T = Tobacco. (PNG) [file pone.0126259.s001.png]

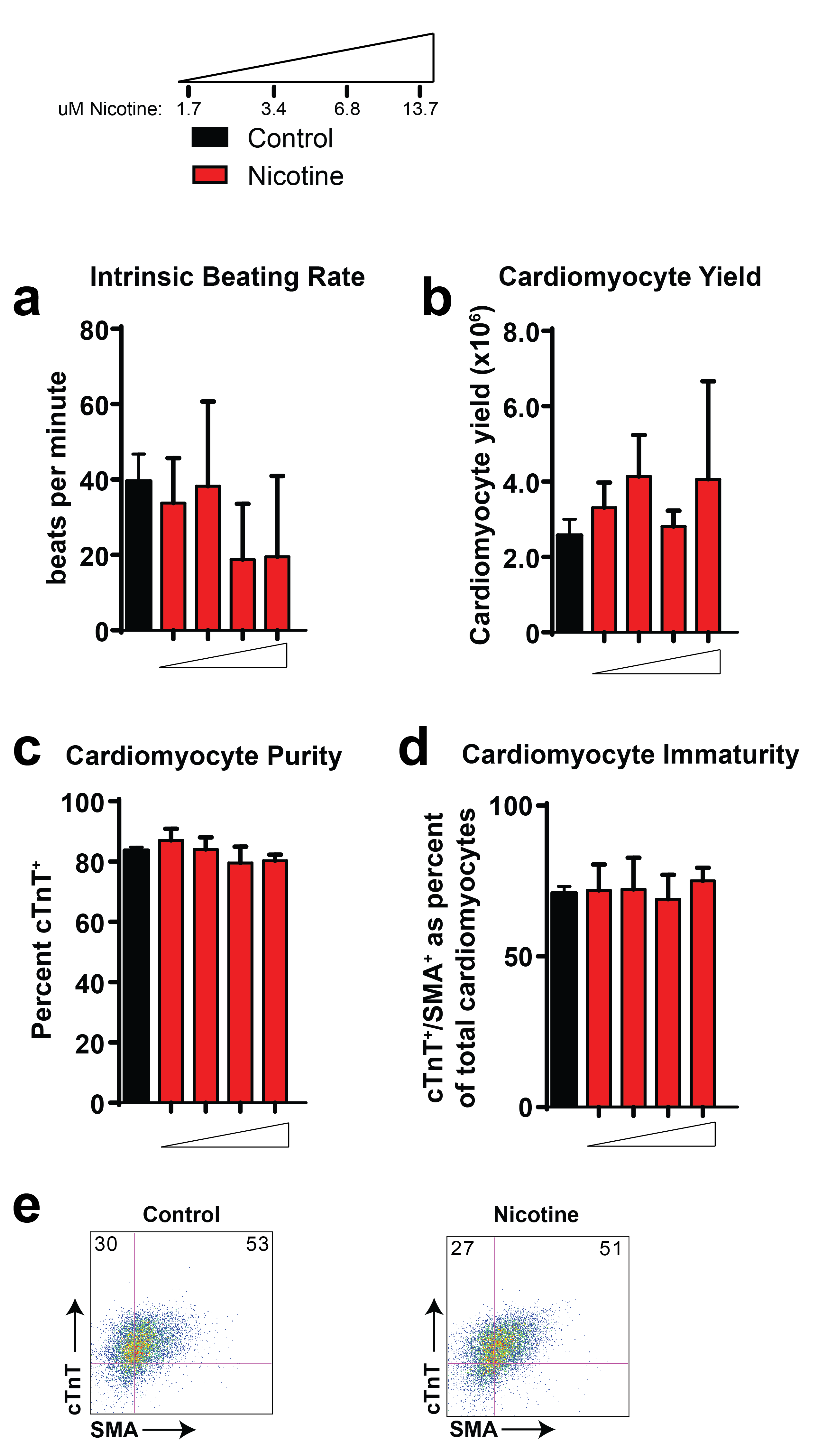

Supplement: S2 Fig — Cells were differentiated into the cardiac lineage and assayed for different markers of cardiomyocyte development at day 14 including intrinsic beating rate (a), cardiomyocyte yield (b), cardiomyocyte purity based on percent cTnT+ cells (c), and cardiomyocyte immaturity based on co-expression of cTnT and SMA (d). (e) Representative flow plots. (PNG) [file pone.0126259.s002.png]

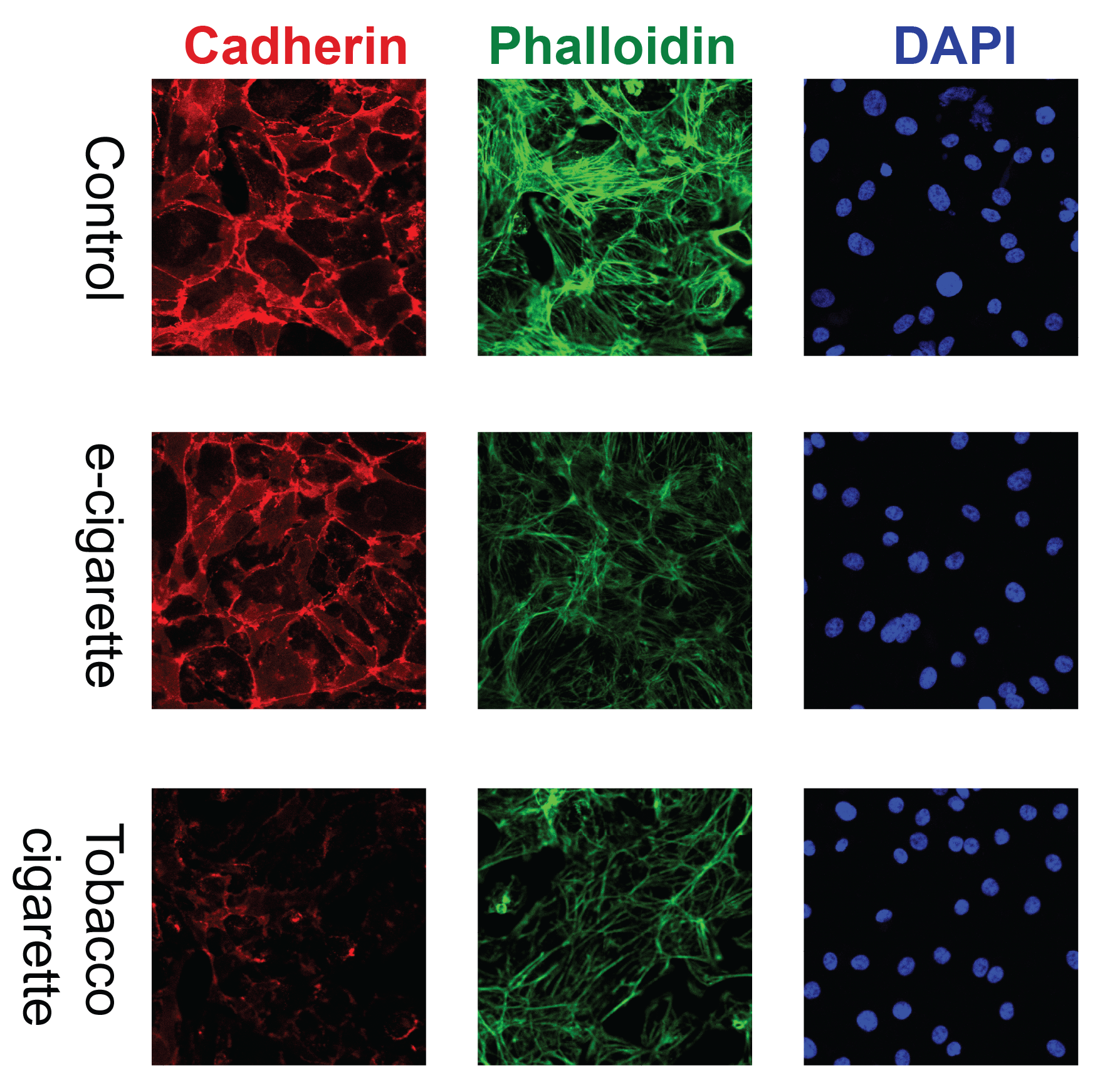

Supplement: S3 Fig — Images are an expanded version of those shown in Fig 5d. (PNG) [file pone.0126259.s003.png]

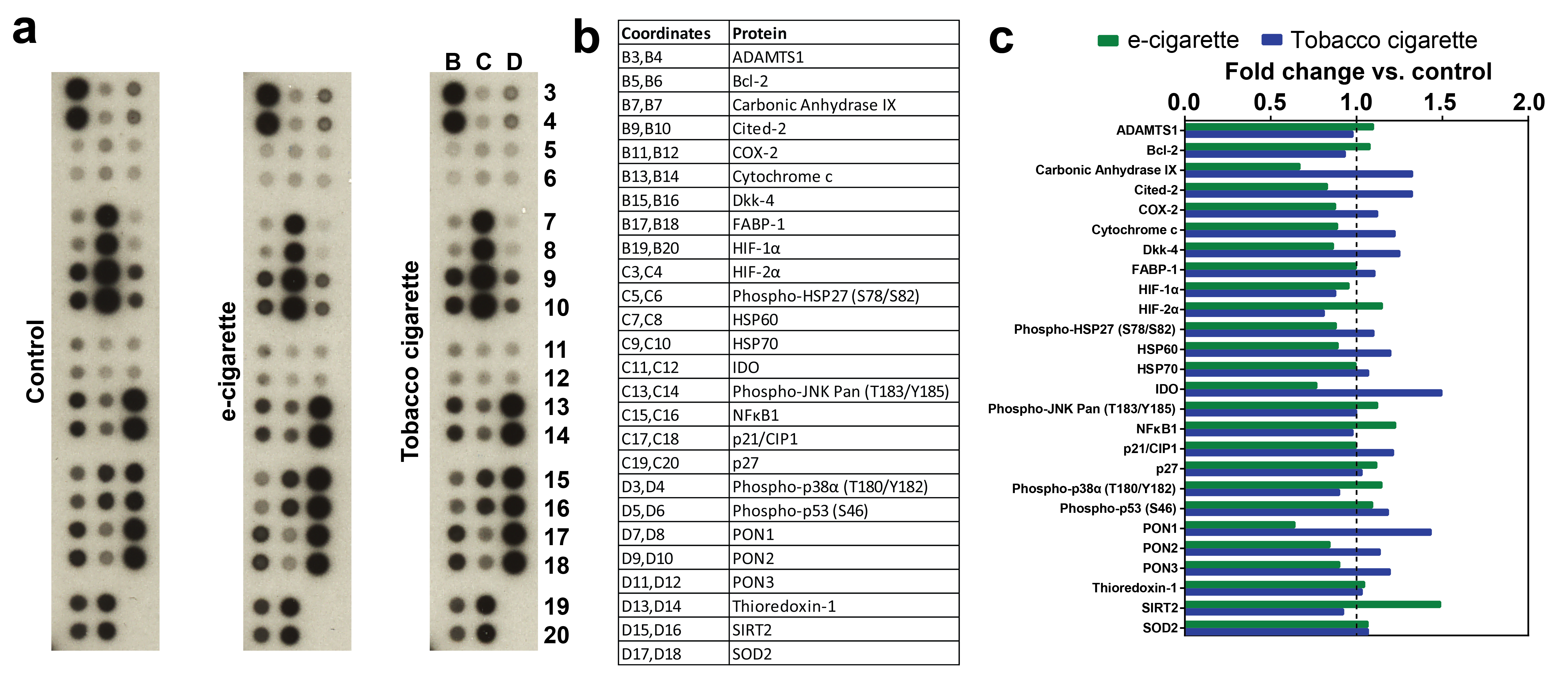

Supplement: S4 Fig — (a) Raw array results used for quantification of 26 cell stress proteins in control, e-cigarette, and tobacco cigarette treated samples. (b) Coordinates for identification of proteins represented in (a). (c) Quantification of mean pixel intensity for each cell stress protein in e-cigarette and tobacco cigarette treated samples compared to control (dotted line). (PNG) [file pone.0126259.s004.png]
