# Supplementary material for: Cardiac Development in Zebrafish and Human Embryonic Stem Cells Is Inhibited by Exposure to Tobacco Cigarettes and E-Cigarettes
Source: PLoS One. 2015 May 15;10(5):e0126259. doi: 10.1371/journal.pone.0126259 (PMC4433280; doi:10.1371/journal.pone.0126259)
Supplement: S1 Table — (DOCX) [file pone.0126259.s005.docx]

**Supporting Information Table 1.**

**Primers used for quantitative RT-PCR: Human**

|  |  |  |
| --- | --- | --- |
| **Gene Name** | **Forward Primer** | **Reverse Primer** |
| HPRT | TGACACTGGCAAAACAATGCA | GGTCCTTTTCACCAGCAAGCT |
| TNNT2 | TTCACCAAAGATCTGCTCCTCGCT | TTATTACTGGTGTGGAGTGGGTGTGG |
| T | CAAATCCTCATCCTCAGTTTG | GTCAGAATAGGTTGGAGAATTG |
| MYH6 | CAAGTTGGAAGACGAGTGCT | ATGGGCCTCTTGTAGAGCTT |
| MYH7 | GGGCAACAGGAAAGTTGGC | ACGGTGGTCTCTCCTTGGG |
| MYL7 | TCCAACGTCTTTTCCATGTT | TCTGTCCCATTGAGCTTCTC |
| MYL2 | CGTTCGGGAAATGCTGACCACGC | AGTCCAAGTTTCCAGTCACGTCAG |
| NODAL | TGGAGGTGGGATGAAGTCACCTAT | AACCCAGCCTGAGGCAATGAGATT |
| DKK1 | AACAGCTATCCAAATGCAG | TCACAGGGGAGTTCCATAAA |
| WNT5a | TAGCAGCATCAGTCCACAAA | CAAAACACGGCATCTCTCTT |
| NKX2.5 | CCAAGGACCCTAGAGCCGAA | ATAGGCGGGGTAGGCGTTAT |
| CACNA1C | CAGAGGCTACGATTTGAGGA | GCTTCACAAAGAGGTCGTGT |
| ATP2A2 | ATGACAACCCACTGAGAAGAGAA | CGAAGGTCAGATTGGTCTCATATTT |
| GATA4 | ACACCCCAATCTCGATATGTTTG | GTTGCACAGATAGTGACCCGT |
| GSC | GAGGAGAAAGTGGAGGTCTGGTT | CTCTGATGAGGACCGCTTCTG |
| MESP1 | TCGAAGTGGTTCCTTGGCAGAC | CCTCCTGCTTGCCTACAAAGTGTC |
| EOMES | CACATTGTAGTGGGCAGTGG | CGCCACCAAACTGAGATGAT |
| WNT8a | GCAGAGGCGGAACTGATCTT | CGACCCTCTGTGCCATAGATG |
| WNT3a | AACTACGTGGAGATCATGCCC | GACTCCCTGGTAGCTTTGTC |

**Primers used for quantitative RT-PCR: Zebrafish**

| **Gene Name** | **Forward Primer** | **Reverse Primer** |
| --- | --- | --- |
| b-actin | AAGCAGGAGTACGATGAGTC | TGGAGTCCTCAGATGCATTG |
| cmlc2 | GGAGAGAAGCTCAATGGCACA | GTCATTAGCAGCCTCTTGAACTCA |
| tnnt2 | GTCTGCACTTCGGCGGTTACA | GCACAGCATTCACTTCCTGA |
| nkx2.5 | GGGATGGTAAACCGTGTCTG | TTGCTGTTGGACTGTGAAGG |
| gata4 | GGCTCCTCTGAAGGTCAGTC | CAGGCTGTTCCACACTTCAC |
| mef2ca | AGAAGAGCGTTTTCGGACTG | GTTCCTCAGCGCAGTCTTTC |
| cacna1c | CACCCAATGCTAACGGAAAC | GCACTCCATGTCCTCATCCT |
| ryr2b | TCCAGCAGGACACTTGACAC | CCAGATCCTCTCTGGGAACA |
| cx43 | TTCAAGTGCAATACCCAGCA | GGCCTTCAGCTCCTCTTCTT |
